# Comparison of 3C4L and 2C3L radiofrequency ablation strategies for persistent atrial fibrillation: procedural profile and 2-year outcomes

**DOI:** 10.3389/fcvm.2025.1591569

**Published:** 2026-02-18

**Authors:** Pengcheng Zhao, Jie Yuan, Zhongyi Jin, Yuhan Zhao, Ting Zhang

**Affiliations:** 1 Department of Cardiovascular Medicine 3, Dongzhimen Hospital, Beijing University of Traditional Chinese Medicine, Beijing, China; 2 Department of Cardiovascular Medicine 4, Dongzhimen Hospital, Beijing University of Traditional Chinese Medicine, Beijing, China

**Keywords:** arrhythmia disease, atrial fibrillation, quantitative parameters of myocardial motion, radiofrequency ablation, serum biomarkers

## Abstract

**Objective:**

To investigate the effects of 3C4L vs. 2C3L radiofrequency ablation strategies on quantitative myocardial motion parameters and serum biomarkers in patients with persistent atrial fibrillation.

**Methods:**

A retrospective study was conducted on 105 patients with persistent atrial fibrillation who underwent radiofrequency ablation treatment at our hospital between March 2023 and November 2024. All patients' data were obtained from the medical record system. Patients were divided into two groups based on the ablation strategy employed: a 2C3L group (*n* = 50, two circumferential lesions plus three linear lesions) and a 3C4L group (*n* = 55, three circumferential lesions plus four linear lesions). The choice of ablation strategy was determined by the treating physician based on atrial fibrillation characteristics, left atrial size, and institutional experience during the study period. Clinical baseline data, perioperative related indexes, quantitative myocardial exercise parameters, and serum biomarkers were compared between the two groups.

**Results:**

There was no statistically significant difference in circumferential pulmonary vein isolation ablation time, cavotricuspid isthmus ablation time, and left atrial apical line ablation time between the 3C4L group and the 2C3L group (*P* > 0.05). However, the 3C4L group had significantly longer total procedure time (172.06 ± 39.33 vs. 156.10 ± 36.37 min, *P* = 0.034), mitral isthmus ablation time (14.58 ± 2.78 vs. 10.21 ± 2.96 min, *P* < 0.001), X-ray exposure time (7.05 ± 1.87 vs. 2.60 ± 0.42 min, *P* < 0.001), and greater saline perfusion volume (1,197.43 ± 184.47 vs. 1,072.33 ± 57.42 mL, *P* < 0.001) compared to the 2C3L group. Before treatment, there were no significant differences between groups in quantitative myocardial motion parameters and serum biomarker indexes (*P* > 0.05). After treatment, the 3C4L group showed significantly better improvement in quantitative myocardial motion parameters and serum biomarker indexes compared to the 2C3L group (all *P* < 0.05). The total incidence rate of complications was not significantly different between the 3C4L group (20.00%, 11/55) and the 2C3L group (16.00%, 8/50) (*χ*^2^ = 0.575, *P* = 0.448). At 24 months, Kaplan–Meier analysis demonstrated significantly higher arrhythmia-free survival in the 3C4L group compared to the 2C3L group (50.0% vs. 30.0%, log-rank *P* = 0.018; hazard ratio 0.52, 95% confidence interval: 0.30–0.90).

**Conclusion:**

Compared with the 2C3L radiofrequency ablation strategy, the 3C4L strategy, while requiring longer procedure time, does not significantly increase complication rates and demonstrates superior clinical efficacy with better improvement in cardiac function and long-term arrhythmia-free survival. These findings should be interpreted cautiously given the retrospective, non-randomized study design and require validation through prospective randomized controlled trials.

## 1 Introduction

Atrial fibrillation (AF), as a common arrhythmia disease, refers to the loss of regular and orderly atrial electrical activity, replaced by rapid and disorderly fibrillation waves, usually manifested as irregular and rapid heart rate, palpitations, dizziness, chest tightness, and fatigue, which are common symptoms of patients with atrial fibrillation, and some patients may also experience syncope, polyuria, and so on. In severe cases, thromboembolism, heart failure, and other complications can occur, posing a serious threat to patients' health and life safety (1, 2). The incidence of AF is on the rise globally, and the number of AF patients worldwide has exceeded 33 million and is expected to reach 150 million by 2050, making it a major public health problem (3, 4). Beyond symptom control, catheter ablation has emerged as an important therapeutic option with demonstrated benefits on hard clinical outcomes. Recent systematic reviews and meta-analyses of randomized controlled trials have established high-quality evidence demonstrating that catheter ablation significantly reduces the risk of stroke and improves mortality outcomes, particularly in patients with heart failure and reduced ejection fraction (5–8). In patients with heart failure, catheter ablation has been shown to reduce all-cause mortality and improve cardiac function compared to medical therapy alone (5). Furthermore, catheter ablation has demonstrated a significant reduction in stroke risk compared to medical management, with benefits observed across different AF subtypes (8). These findings underscore the role of catheter ablation not merely as a rhythm control strategy but as an intervention capable of improving major cardiovascular outcomes. Therefore, exploring effective AF treatment strategies is important for improving patient prognosis and reducing the social healthcare burden.

**Figure 1:**
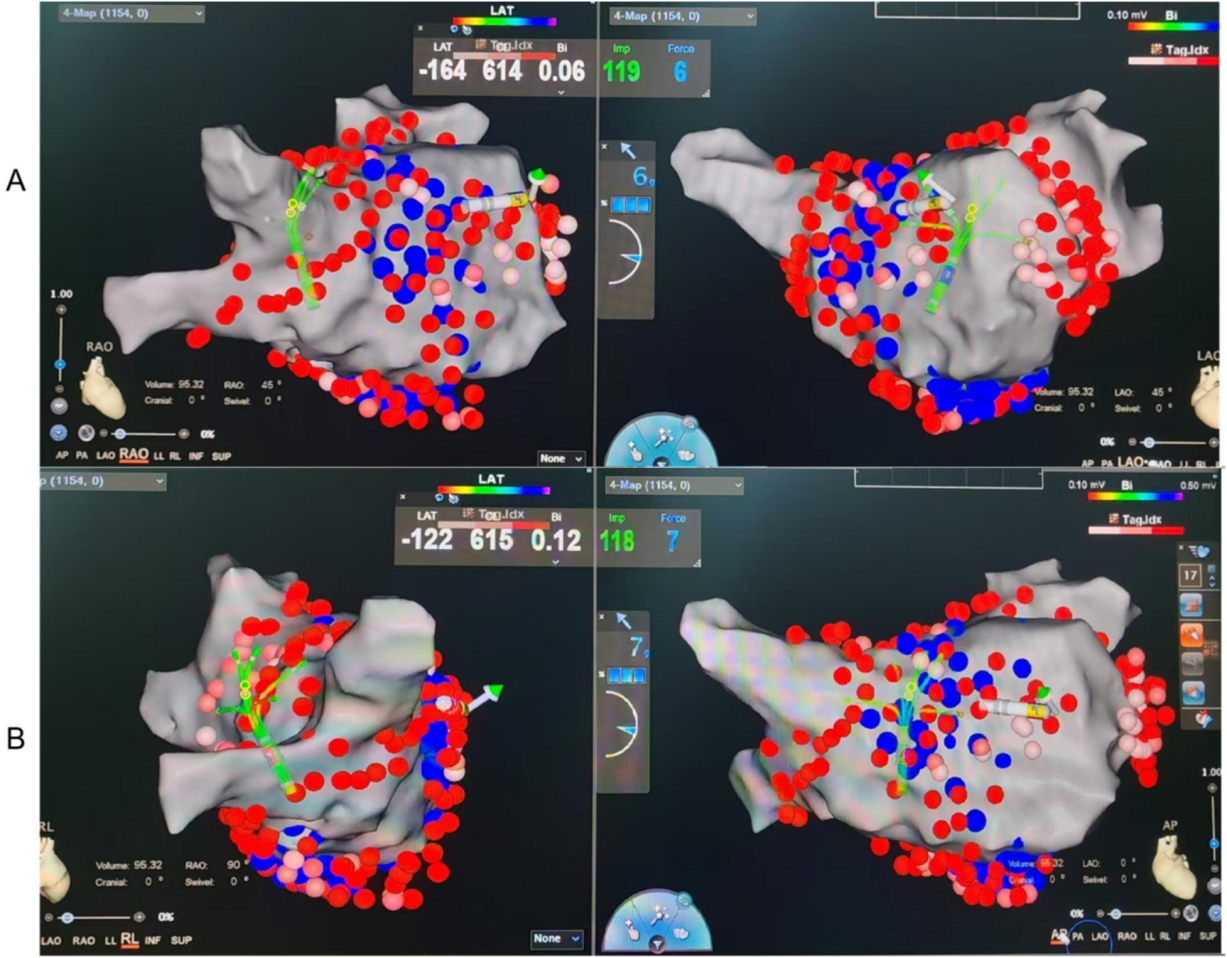
Representative CARTO electroanatomical maps illustrating lesion sets for the two ablation strategies. **(A)** 2C3L strategy: Two circumferential pulmonary vein isolations (CPVI) plus three linear lesions (roof line, mitral isthmus line, and cavotricuspid isthmus line). **(B)** 3C4L strategy: Three circumferential isolations (bilateral PV antra plus posterior wall “box” isolation) plus four linear lesions (roof line, posterior wall floor line, mitral isthmus line, and cavotricuspid isthmus line). Red lines indicate radiofrequency ablation lesions. LA, left atrium; PV, pulmonary vein; LIPV/RIPV, left/right inferior PV; LSPV/RSPV, left/right superior PV; LAA, left atrial appendage; CTI, cavotricuspid isthmus; MI, mitral isthmus; MV, mitral valve.

The 2C3L radiofrequency ablation strategy consists of three ablation routes around the right and left atriums (3L), as well as the connection of the two transverse ablation lines (2C), which blocks abnormal electrical signals by creating fewer ablation lines and has a relatively simple procedure with low procedure time and risk but has some risk in reducing recurrence rates. The 3C4L radiofrequency ablation strategy is a complex and comprehensive arrhythmia treatment method that is characterized by the creation of multiple linear ablation lesions within the heart to block abnormal electrical signal conduction, including four ablation lines around the left and right atria (4L), as well as three transverse ablation lines connecting the left and right atria (3C), aiming to achieve bidirectional blockade. The 3C4L strategy is based on the 2C3L strategy and adds the isolation line of the posterior wall of the left atrium. Figures 1A,B give a step-by-step layout. Several studies have shown that the 3C4L strategy can eliminate AF foci more comprehensively, reduce the recurrence rate, and improve the quality of life of patients, but the operation of this strategy is relatively complex, the procedure time is longer, and it may increase the complications (9, 10).

## 2 Materials and methods

### 2.1 Statement of ethics

This study was approved by the Dongzhimen Hospital of Beijing University of Traditional Chinese Medicine Institutional Review Board and Ethics Committee (Approval No. 2021-KL-034-01). Given that this study was retrospective and only deidentified patient data were used, informed consent was not required as there were no risks or adverse effects to patients. This waiver is in line with regulatory and ethical guidelines related to retrospective studies.

Ethics statement: Our research is in strict accordance with and adheres to the Declaration of Helsinki. The study was approved by the Ethics Committee of Dongzhimen Hospital of Beijing University of Traditional Chinese Medicine. The study is retrospective; no trial ID is mandated under local regulations.

### 2.2 Study design

This retrospective analysis studied 105 patients with persistent atrial fibrillation who underwent radiofrequency ablation at our hospital between March 2023 and November 2024, with all patients' data coming from the medical records system. Patients were assigned to either the 2C3L group (*n* = 50) or the 3C4L group (*n* = 55) based on the ablation strategy employed during their treatment. The selection of ablation strategy was determined by the treating electrophysiologist based on multiple clinical factors including AF duration, left atrial size, AF burden, previous ablation history, and the evolving institutional experience and preferences during the study period. During the earlier phase of the study period (March 2023 to June 2023), the 2C3L approach was primarily used as it represented the standard institutional practice. As institutional experience evolved and evidence emerged supporting more extensive ablation strategies, the 3C4L approach was increasingly adopted from July 2023 onward for patients with persistent AF, particularly those with larger left atrial dimensions and longer AF duration. The decision was made on a case-by-case basis by experienced electrophysiologists, considering individual patient characteristics and procedural feasibility.

### 2.3 Inclusion criteria

Patients were eligible if they had a valid indication for AF ablation and no contraindication to the procedure; the following were the key exclusions: (1) patients who combined with severe organic thyroid abnormality and renal insufficiency; (2) patients who combined with malignant tumors and other consumptive diseases; (3) patients with survival life expectancy <1 year; (4) previous superior vena cava stenosis, abnormal sinus node function, and after pacemaker implantation; (5) transthoracic cardiac ultrasound showed that the anterior and posterior diameters of the left atrium were greater than 55 mm; (6) there was a contraindication to anticoagulation; and (7) women during pregnancy and lactation.

### 2.4 Treatment

#### 2.4.1 Preoperative preparation

All patients routinely received oral warfarin or novel oral anticoagulants for at least 3 weeks before surgery. Patients taking warfarin maintained an INR of 2.0–3.0, while those taking novel oral anticoagulants discontinued them on the day of surgery. All antiarrhythmic drugs (AADs) except amiodarone were discontinued for at least five half-lives before surgery, and amiodarone was discontinued for at least 8 weeks prior to surgery. Patients fasted for at least 8 h before surgery and were placed in a supine position on the operating table. General anesthesia was administered, and pericardial cavity was monitored using transthoracic ultrasound preoperatively, postoperatively, and as necessary during the procedure.

#### 2.4.2 Electrophysiological examination and parameter setting

A 10-pole fixed-curve diagnostic electrophysiology catheter (Biosense-Webster Inc.) was placed in the coronary sinus (CS) via the patient's right axillary vein. Interatrial septal puncture was performed via the right femoral vein. Following successful puncture, left atrial and pulmonary venography were performed, and 100 U/kg of unfractionated heparin was administered, with an additional 1,000 U administered every hour thereafter. A high-density mapping electrode catheter (PENTARAY® Catheter, Biosense-Webster Inc.) was then delivered via the interatrial septal puncture sheath. The left atrium was anatomically reconstructed under three-dimensional guidance using the pulmonary veins (CARTO, Biosense-Webster Inc.) to accurately localize the pulmonary vein openings and vestibular locations. The septum was punctured a second time, and a saline-irrigated ablation catheter (Thermocool Smarttouch SF, Biosense-Webster) was delivered to the left atrium to perform point-by-point ablation using a power-controlled mode under saline irrigation. The preset ablation power was 50 W, the preset temperature was 41°C, contact force was maintained between 10 and 15 g, the saline infusion rate was 15 mL/min during ablation and 2 mL/min between ablations, and ablation indexes (AIs) were set at 450–500 for the pulmonary vein vestibule, 350–400 for the posterior wall, 400–420 for the roof and floor of the left atrium, the superior vena cava, and the cavotricuspid isthmus (CTI), respectively, and 500–550 for the mitral isthmus.

#### 2.4.3 Radiofrequency ablation strategy

Representative CARTO electroanatomical maps illustrating the lesion sets for both ablation strategies are shown in Figure 1. Patients undergoing the 2C3L radiofrequency ablation strategy first underwent circumferential pulmonary vein isolation (CPVI), followed by connection of bilateral pulmonary veins to form a roof line, then ablation of the mitral isthmus (MI) from the mitral annulus to the left inferior pulmonary vein orifice, and ablation of the CTI from the tricuspid annulus at the 6 o'clock position to the inferior vena cava. Patients were observed for reversion to sinus rhythm. If atrial fibrillation persisted or converted to atrial tachycardia, electrical cardioversion was performed. Subsequently, bidirectional block was verified for all ablation lines. If not achieved, additional ablation was performed.

In patients undergoing the 3C4L radiofrequency ablation strategy, box isolation of the posterior wall of the left atrium (POWI) was performed in addition to CPVI, including ablation of the roof and floor lines of the left atrium and endocardial ablation of the MI. Patients were observed for restoration of sinus rhythm. If sinus rhythm was not restored, synchronized direct current cardioversion (150 J biphasic) was performed. Subsequently, bidirectional block was verified for all ablation pathways, and necessary additional ablation including ethanol infusion into the vein of Marshall (EI-VOM) was performed. Finally, CTI and superior vena cava electrical isolation (SVCI) were performed and verified.

#### 2.4.4 Surgical endpoints

The primary surgical endpoint was defined as completion of all ablation lines of the 2C3L and 3C4L strategies in sinus rhythm and achievement of bidirectional, irreversible block of all ablation lines in a safe manner. The validation methods were as follows: (1) CPVI: absence of potentials or only isolated potentials in the pulmonary vein verified in sinus rhythm; bidirectional block between the pulmonary vein and the left atrium was also verified by mapping or ablation catheter recording or pacing. (2) MI block: When the left atrium was stimulated, excitation conducted from the proximal CS to the distal CS via the lateral wall of the left atrium, or the conduction time of excitation from the lateral wall of the left atrium to the ablation line was longer when pacing distally in the CS compared to proximal CS pacing, confirming MI block. (3) CTI block: When pacing the right atrial free wall, the order of excitation conduction was from the septum to the ablation line, whereas when pacing the proximal CS, the order of excitation conduction was from the right atrial free wall to the ablation line. (4) SVCI: disappearance of superior vena cava intracardiac potential or dissociation of the superior vena cava potential from the atrial intracardiac potential. (5) Left atrial (LA) roof line: In sinus rhythm, the order of atrial excitation was demonstrated from superior to inferior on the anterior wall of the left atrium and from inferior to superior on the posterior wall. (6) POWI: (a) Bidirectional block in the roof line; (b) voltage in the posterior wall of the left atrium <0.1 mV; and (c) entrance and exit block in the isolated region. All validation procedures were completed 20 min later. If conduction was present, additional ablation was performed. The secondary surgical endpoint was failure to achieve bidirectional block in the ablation pathway after completion of all planned ablation lines.

### 2.5 Clinical baseline data

Age, gender (male/female), duration of atrial fibrillation, comorbidities (diabetes mellitus, hypertension, coronary artery disease) (presence/absence), cardiac insufficiency (presence/absence), history of smoking (presence/absence), and history of alcohol consumption (presence/absence) were collected from both groups.

### 2.6 Surgery-related indicators

Total operation time, CPVI ablation time, MI ablation time, CTI ablation time, LA top line ablation time, X-ray exposure time, and saline infusion volume were collected and compared between the two groups.

### 2.7 Serum biomarkers

Five milliliters of fasting venous blood were collected from patients in the morning and centrifuged using a centrifuge (model: LL900, manufacturer: Luoyang Hongshi Machinery Equipment Co., Ltd.) at 3,000 r/min for 10 min to separate serum, which was then stored at −70°C until analysis. Serum amino-terminal B-type brain natriuretic peptide precursor (NT-ProBNP) and monocyte chemotactic protein 1 (MCP-1) levels were detected by enzyme-linked immunosorbent assay, serum uric acid (UA) levels were detected by chemiluminescence, and all operations were performed according to the manufacturer's instructions (Shanghai Enzymatic Union Biotechnology Co., Ltd.). N-acetylneuraminic acid (Neu5Ac) and indoxyl sulfate (IS) were measured by liquid chromatography-tandem mass spectrometry.

### 2.8 Quantitative parameters of myocardial exercise

GE Vivid E95 diagnostic ultrasound machine with M5S probe (frequency 1.5–4.6 MHz) and 4 V probe (frequency 1.5–4.0 MHz) was applied. According to the guidelines of the American Society of Echocardiography, five consecutive cardiac cycles were measured and averaged, including the left atrial anteroposterior diameter (LAD), left atrial volume index (LAVI), left atrial maximum volume (LAVmax), left atrial minimum volume (LAVmin), and the left ventricular ejection fraction (LVEF), which was measured by the modified biplane Simpson method.

### 2.9 Incidence of complications

Perioperative complications such as fever, pericardial effusion, heart failure, pericardial tamponade, atrial stiffness syndrome, and stroke were recorded in all patients and the total incidence was statistically compared.

### 2.10 Follow-up and outcome assessment

All patients were followed up at 3, 6, 12, 18, and 24 months after ablation through outpatient visits or telephone contact. Arrhythmia recurrence was defined as any documented atrial tachyarrhythmia (atrial fibrillation, atrial flutter, or atrial tachycardia) lasting more than 30 s occurring beyond a 3-month blanking period after the ablation procedure. Recurrence was documented through 12-lead electrocardiography, 24 h Holter monitoring, or event-triggered recordings. Arrhythmia-free survival was calculated from the end of the 3-month blanking period to the first documented recurrence or last follow-up.

### 2.11 Statistical analysis

This article uses SPSS 25.0 statistical software to calculate the data, the count data are expressed by [*n*(%)], and Fisher's exact test was used when any expected cell count was less than 5, otherwise the *χ*^2^ test was adopted; the measurement data were tested by the Shapiro–Wilk method to be in line with the normal distribution, and are expressed by $\left(\bar{x}\pm s\right)$, the comparison between the two groups is made by the independent samples *t*-test, and the comparison within a group is made by the paired samples *t*-test. Kaplan–Meier survival analysis was performed to estimate arrhythmia-free survival, and the log-rank test was used to compare survival curves between groups. Cox proportional hazards regression analysis was conducted to calculate hazard ratios (HR) with 95% confidence intervals (CI) for arrhythmia recurrence, adjusting for potential confounders including age, sex, AF duration, left atrial diameter, and LVEF. The difference of *P* < 0.05 is considered to be statistically significant.

### 2.12 Comparison of clinical baseline data between the two groups of patients

There was no statistically significant difference between the two groups in terms of age, sex, AF duration, cardiovascular comorbidities (including hypertension, diabetes mellitus, coronary artery disease, heart failure, and stroke/TIA history), lifestyle factors (smoking and alcohol consumption), and baseline echocardiographic parameters (LAD, LAVI, LAVmax, LAVmin, and LVEF) (all *P* > 0.05, Table 1). These findings indicate comparable baseline characteristics between the two treatment groups.

**Table 1:** Comparison of clinical baseline data between the two groups.

Index	3C4L group (*n* = 55)	2C3L group (*n* = 50)	*t/χ* ^2^	*P*
Age (years, $\bar{x}\pm s$)	59.33 ± 9.74	61.53 ± 9.74	1.156	0.250
Sex (*n*, %) male	30 (54.55)	25 (50.00)	0.217	0.641
Female	25 (45.45)	25 (50.00)		
Duration of atrial fibrillation (months, $\bar{x}\pm s$)	12.88 ± 3.78	12.67 ± 3.82	0.283	0.778
Complication (*n*, %) have	12 (21.82)	10 (20.00)	0.052	0.819
No	43 (78.18)	40 (80.00)		
Cardiac insufficiency (*n*, %) have	15 (27.27)	13 (26.00)	0.022	0.883
No	40 (72.73)	37 (76.00)		
Smoking history (*n*, %) have	25 (45.55)	21 (42.00)	0.127	0.722
No	30 (54.55)	29 (58.00)		
Drinking history (*n*, %) have	19 (34.55)	14 (28.00)	0.521	0.471
No	36 (65.45)	36 (72.00)		

### 2.13 Comparison of surgery-related indexes between the two groups of patients

Perioperative-related indexes are assessment indexes of the process of each step of surgery, effectively reflecting the efficiency of surgery. There was no statistically significant difference between the 3C4L group and the 2C3L group in terms of CPVI ablation time (20.06 ± 3.30 vs. 20.33 ± 4.67 min), CTI ablation time (11.35 ± 4.04 vs. 12.14 ± 3.83 min), and LA apical line ablation time (3.41 ± 1.25 vs. 3.45 ± 0.96 min) (*P* > 0.05). However, in the 3C4L group, the total operation time (172.06 ± 39.33 vs. 156.10 ± 36.37 min), MI ablation time (14.58 ± 2.78 vs. 10.21 ± 2.96 min), X-ray exposure time (7.05 ± 1.87 vs. 2.60 ± 0.42 min), and the volume of saline irrigation (1,197.43 ± 184.47 vs. 1,072.33 ± 57.42 mL) were higher than the 2C3L group. The difference was statistically significant (*P* < 0.05), thus indicating that compared to the 2C3L radiofrequency ablation strategy, the 3C4L radiofrequency ablation strategy had a longer procedure-related time and a high saline perfusion volume, as presented in Table 2.

**Table 2:** Comparison of surgery-related indexes between the two groups $\left(\bar{x}\pm s\right)$.

Index	3C4L group (*n* = 55)	2C3L group (*n* = 50)	*t*	*P*
Total operating time (min)	172.06 ± 39.33	156.10 ± 36.37	2.152	0.034
CPVI ablation time (min)	20.06 ± 3.30	20.33 ± 4.67	0.345	0.731
MI ablation time (min)	14.58 ± 2.78	10.21 ± 2.96	7.800	<0.001
CTI ablation time (min)	11.35 ± 4.04	12.14 ± 3.83	1.026	0.307
Ablation time of LA top line (min)	3.41 ± 1.25	3.45 ± 0.96	0.183	0.856
X-ray exposure time (min)	7.05 ± 1.87	2.60 ± 0.42	16.447	<0.001
Brine perfusion (mL)	1,197.43 ± 184.47	1,072.33 ± 57.42	4.595	<0.001

CPVI, circumferential pulmonary vein isolation; MI, mitral isthmus; CTI, tricuspid isthmus; LA, left atrium.

### 2.14 Comparison of serum biomarkers between the two groups of patients

Various serum biomarkers are closely related to disease recurrence and can effectively assess the effect of radiofrequency ablation strategy treatment. Before treatment, NT-ProBNP (973.45 ± 103.87 vs. 971.17 ± 99.23 ng/L), MCP-1 (246.33 ± 14.56 vs. 245.02 ± 14.21 pg/mL), UA (461.25 ± 62.35 vs. 460.12 ± 60.66 μmol/L), Neu5Ac (131.72 ± 41.28 vs. 131.29 ± 41.20 ng/mL), and IS (0.69 ± 0.14 vs. 0.67 ± 0.18 μg/mL) indexes were compared and the differences were not statistically significant (*P* > 0.05), as presented in Table 3.

**Table 3:** Comparison of serum biomarkers between the two groups before treatment $\left(\bar{x}\pm s\right)$.

Index	3C4L group (*n* = 55)	2C3L group (*n* = 50)	*t*	*P*
NT-ProBNP (ng/L)	973.45 ± 103.87	971.17 ± 99.23	0.115	0.909
MCP-1 (pg/mL)	246.33 ± 14.56	245.02 ± 14.21	0.466	0.642
UA (μmol/L)	461.25 ± 62.35	460.12 ± 60.66	0.094	0.925
Neu5Ac (ng/mL)	131.72 ± 41.28	131.29 ± 41.20	0.053	0.958
IS (μg/mL)	0.69 ± 0.14	0.67 ± 0.18	0.639	0.525

NT-ProBNP, amino-terminal B-type brain natriuretic peptide precursor; MCP-1, monocyte chemotactic protein 1; UA, uric acid; Neu5Ac, N-acetylneuraminic acid; IS, indole sulfate phenol.

**Figure 2:**
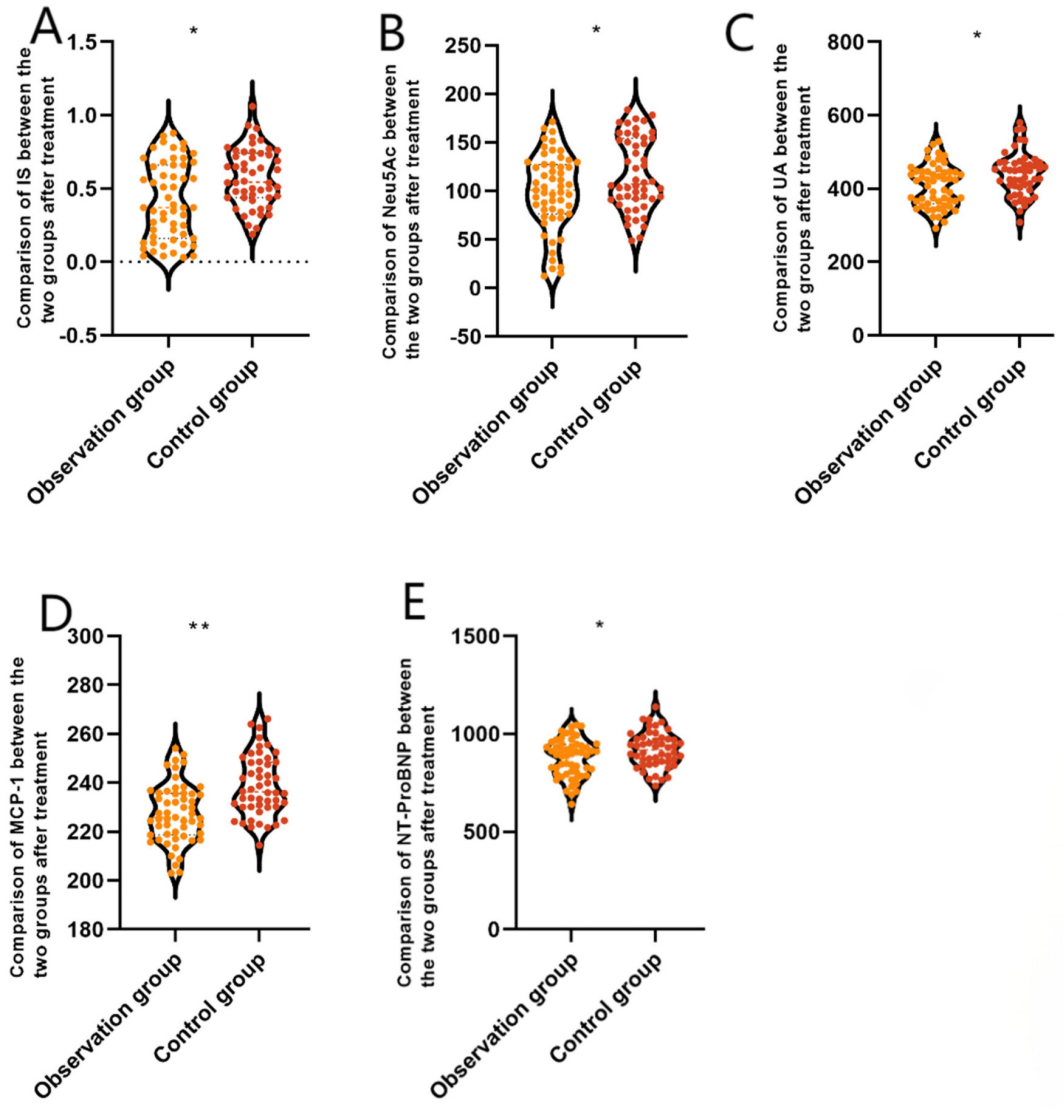
Comparison of serum biomarkers between the two groups after treatment. **(A)** NT-ProBNP, **(B)** MCP-1, **(C)** UA, **(D)** Neu5Ac, and **(E)** IS. Data are presented as mean ± standard deviation. *P < 0.05, **P < 0.001 vs. the control group. NT-ProBNP, amino-terminal B-type brain natriuretic peptide precursor; MCP-1, monocyte chemotactic protein 1; UA, uric acid; Neu5Ac, N-acetylneuraminic acid; IS, indole sulphate phenol.

After treatment, patients in the 3C4L group had NT-ProBNP (874.98 ± 97.48 vs. 918.44 ± 90.37 ng/L), MCP-1 (227.48 ± 12.09 vs. 238.77 ± 12.34 pg/mL), UA (407.09 ± 57.49 vs. 439.41 ± 58.54 μmol/L), Neu5Ac (98.82 ± 39.52 vs. 117.68 ± 37.52 ng/mL), and IS (0.41 ± 0.29 vs. 0.58 ± 0.32 μg/mL) indexes that were lower than those of the 2C3L group, and the difference was statistically significant (*P* < 0.05), which indicated that compared with the 2C3L radiofrequency ablation strategy, the 3C4L radiofrequency ablation strategy had a better efficacy. The results are shown in Figure 2.

### 2.15 Comparison of quantitative myocardial exercise parameters between the two groups of patients

Quantitative myocardial motion parameters are mainly measured by echocardiography, which provides important information about the function and structure of the heart and is used to assess the motion status of the myocardium as well as the overall function of the heart. Prior to the administration, LAD (42.29 ± 3.81 vs. 42.05 ± 4.13 mm), LAVI (48.63 ± 10.93 vs. 48.75 ± 10.52 mL/m^2^), LAVmax (79.17 ± 9.25 vs. 78.53 ± 9.11 mL), LAVmin (52.95 ± 6.82 vs. 52.51 ± 6.34 mL), LVEF (58.23 ± 4.79% vs. 58.35 ± 4.82%) indexes were compared. The difference was not statistically significant (*P* > 0.05), see Table 4.

**Table 4:** Comparison of quantitative parameters of myocardial movement before treatment between the two groups $\left(\bar{x}\pm s\right)$.

Index	3C4L group (*n* = 55)	2C3L group (*n* = 50)	*t*	*P*
LAD (mm)	42.29 ± 3.81	42.05 ± 4.13	0.310	0.757
LAVI (mL/m^2^)	48.63 ± 10.93	48.75 ± 10.52	0.057	0.954
LAVmax (mL)	79.17 ± 9.25	78.53 ± 9.11	0.357	0.722
LAVmin (mL)	52.95 ± 6.82	52.51 ± 6.34	0.341	0.734
LVEF (%)	58.23 ± 4.79	58.35 ± 4.82	0.128	0.899

LAD, left atrial anteroposterior diameter; LAVI, left atrial volume index; LAVmax, left atrial maximum volume; LAVmin, left atrial minimum volume; LVEF, left ventricular ejection fraction.

**Figure 3:**
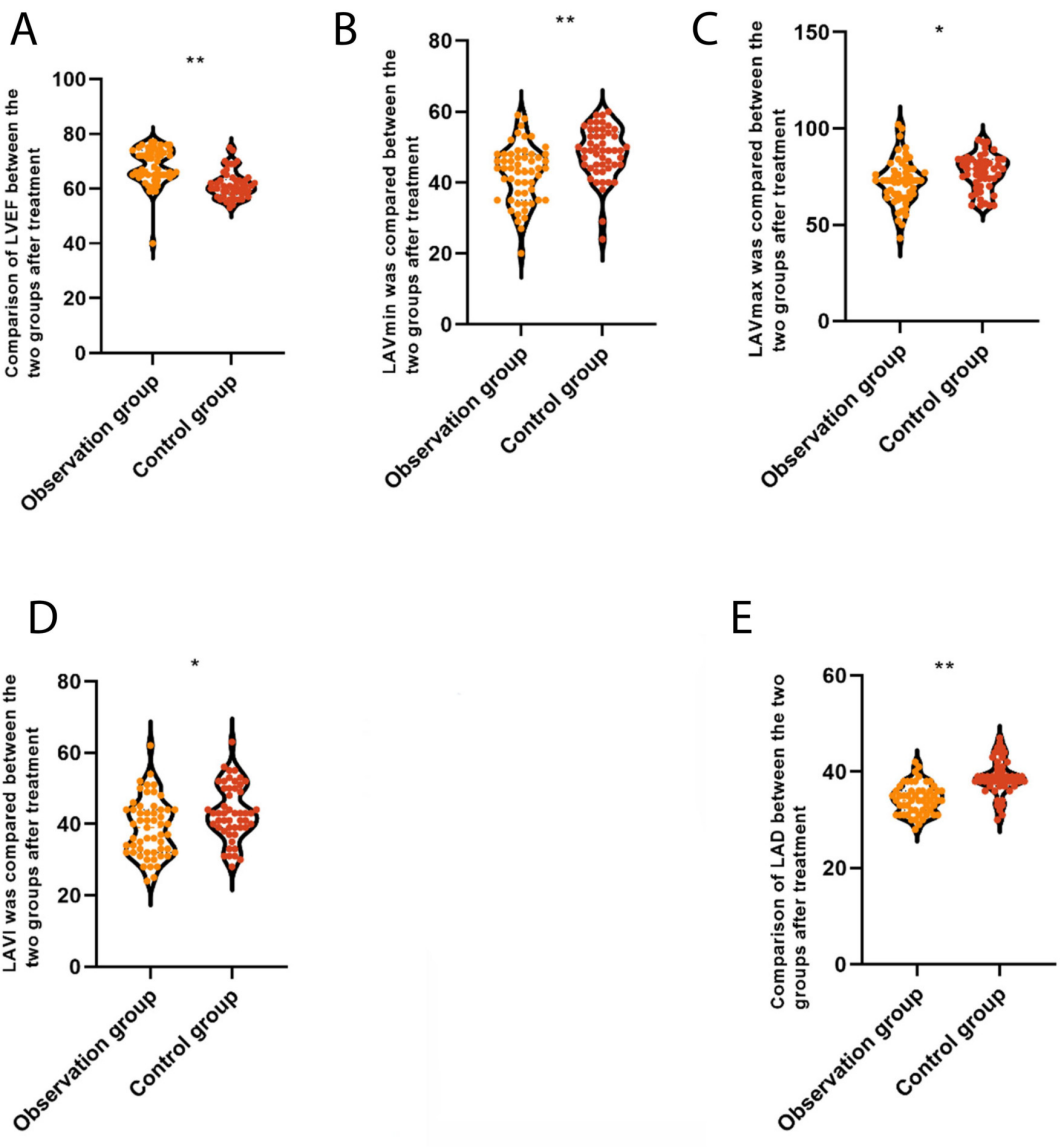
Comparison of quantitative parameters of myocardial movement between the two groups after treatment. **(A)** LAD, left atrial anteroposterior diameter; **(B)** LAVI, left atrial volume index; **(C)** LAVmax, left atrial maximum volume; **(D)** LAVmin, left atrial minimum volume; **(E)** LVEF, left ventricular ejection fraction. *P < 0.05.

After treatment, in the 3C4L group, LAD (34.31 ± 3.12 vs. 38.76 ± 3.76 mm), LAVI (38.92 ± 8.12 vs. 43.26 ± 7.77 mL/m^2^), LAVmax (72.36 ± 11.80 vs. 76.95 ± 10.25 mL), and LAVmin (42.50 ± 8.11 vs. 48.38 ± 7.29 mL) were lower than that of the 2C3L group, but LVEF (68.63 ± 5.37% vs. 61.33 ± 5.02%) was higher than that of the 2C3L group, with a statistically significant difference (*P* < 0.05), which indicated that compared with the 2C3L radiofrequency ablation strategy, the 3C4L radiofrequency ablation strategy had a better effect on the cardiac function, as shown in Figure 3.

### 2.16 Comparison of complications between the two groups of patients

The total complication rate was 20.00% (11/55) in the 3C4L group and 16.00% (8/50) in the 2C3L group. Fisher's exact test was applied for individual complication types due to low expected cell counts, while *χ*^2^ test was used for overall comparison. The difference in total complication rate between groups was not statistically significant (*χ*^2^ = 0.575, *P* = 0.448), which indicated that both 3C4L and 2C3L radiofrequency ablation strategies caused some degree of complications in patients with persistent atrial fibrillation, but there was no significant difference in the comparison of the safety between the two, as presented in Table 5.

**Table 5:** Comparison of complications between the two groups (*n*, %).

Complication	3C4L group (*n* = 55)	2C3L group (*n* = 50)	*P*
Fever	2 (3.64)	2 (4.00)	1.000^a^
Pericardial effusion	1 (1.82)	1 (2.00)	1.000^a^
Heart failure	5 (9.09)	2 (4.00)	0.443^a^
Pericardial tamponade	0 (0.00)	1 (2.00)	0.476^a^
Atrial stiffness syndrome	2 (3.64)	1 (2.00)	1.000^a^
Stroke	1 (1.82)	1 (2.00)	1.000^a^
Total incidence	11 (20.00)	8 (16.00)	0.448^b^

^a^ Fisher's exact test.

^b^ *χ*^2^ test.

### 2.17 Follow-up outcome and survival analysis

**Figure 4:**
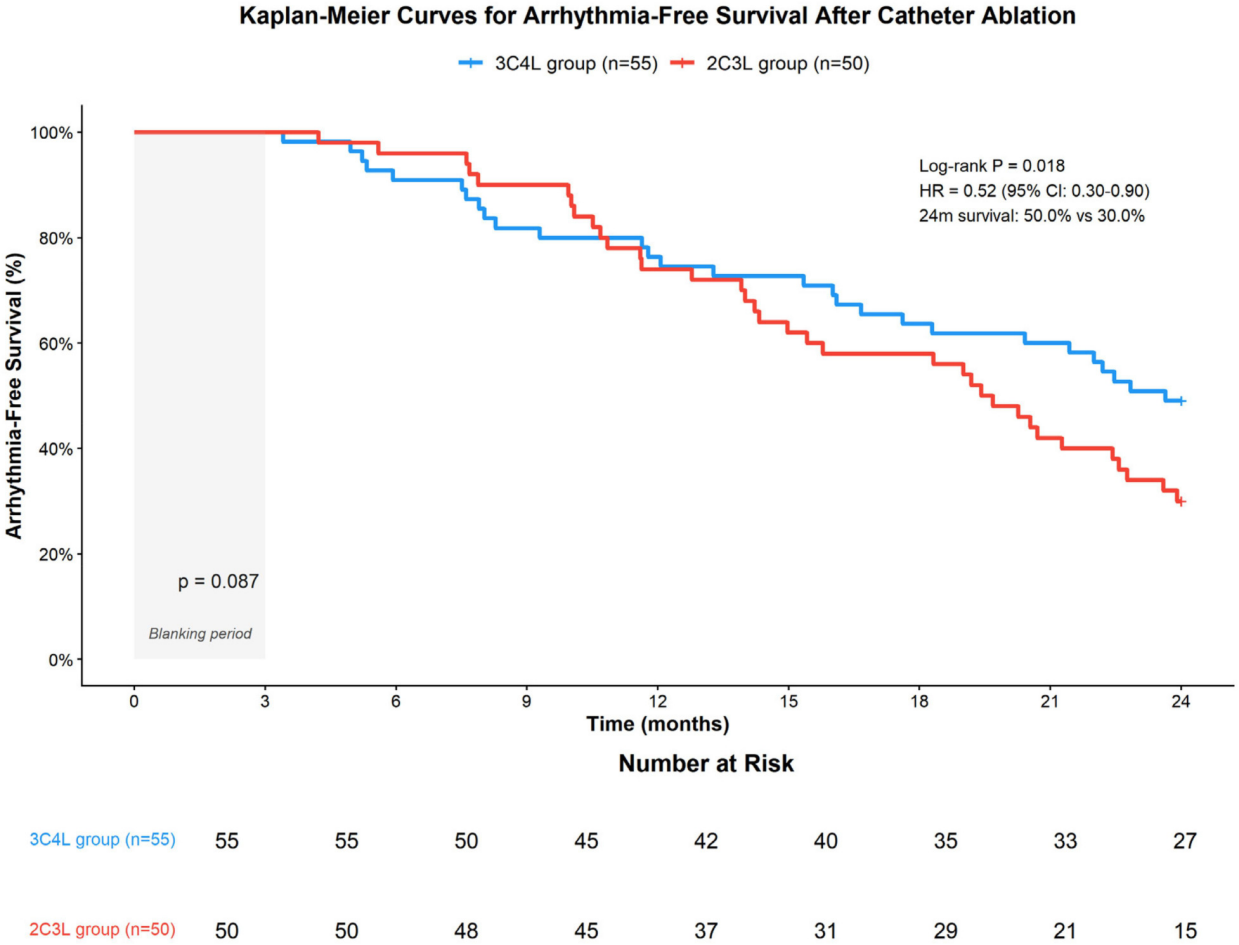
Kaplan–Meier survival curves comparing arrhythmia-free survival between the 3C4L and 2C3L groups over 24 months of follow-up. The 3C4L group demonstrated significantly superior arrhythmia-free survival compared with the 2C3L group (50.0% vs. 30.0% at 24 months, log-rank *P* = 0.018; adjusted hazard ratio 0.52, 95% CI: 0.30–0.90). Numbers at risk are shown below the *x*-axis. The 3-month blanking period is indicated by the shaded area.

During the 24-month follow-up period, complete follow-up data were available for all 105 patients (100% follow-up rate). Kaplan–Meier survival analysis demonstrated significantly higher arrhythmia-free survival in the 3C4L group compared to the 2C3L group at 24 months (50.0% vs. 30.0%, log-rank test *P* = 0.018, Figure 4). Cox proportional hazards regression analysis, after adjusting for age, sex, AF duration, baseline left atrial diameter, and baseline LVEF, confirmed that the 3C4L strategy was independently associated with lower risk of arrhythmia recurrence (adjusted HR 0.52, 95% CI: 0.30–0.90, *P* = 0.020). The median time to first recurrence was 18.3 months (95% CI: 14.2–22.4) in the 3C4L group vs. 12.7 months (95% CI: 9.8–15.6) in the 2C3L group. These findings support the enhanced long-term efficacy of the 3C4L strategy despite its longer procedural duration.

## 3 Discussion

Persistent atrial fibrillation refers to atrial fibrillation that fails to recover on its own for more than 48 h and requires pharmacological or non-pharmacological interventions to recover. Patients experience palpitations and shortness of breath, and are prone to heart failure, while dislodgement of atrial appendage thrombus may lead to pulmonary embolism (11, 12). The ideal goal of AF treatment is restoration of sinus rhythm and normal atrial contraction function while relieving symptoms (13). Although drug therapy represents the traditional treatment approach, its clinical effectiveness for persistent AF remains suboptimal, prompting radiofrequency ablation to emerge as a focal point in persistent AF management.

Cardiac ultrasound parameters including LAD, LAVI, LAVmax, LAVmin, and LVEF serve as important indexes for assessing cardiac structure and myocardial motion function, which are significant in the diagnosis and treatment of persistent AF and can effectively predict AF recurrence (14). Several biomarkers have established associations with AF pathophysiology. NT-ProBNP, secreted by cardiomyocytes and released into the bloodstream during myocardial injury, reflects cardiac stress and dysfunction. UA, the end product of purine metabolism, can induce inflammatory reactions through activation of inflammatory factors and affect atrial remodeling. MCP-1, a chemotactic cytokine, promotes eosinophil and basophil chemotaxis and activation, exacerbating inflammatory responses. Neu5Ac, the predominant form of sialic acid, participates in various pathophysiological processes. IS, derived from dietary protein metabolism by intestinal microbiota, has been associated with the pathogenesis of multiple cardiovascular diseases. All these markers are closely related to the onset, progression, and recurrence of persistent AF (15, 16).

In this study, the 3C4L group demonstrated superior outcomes compared to the 2C3L group in both quantitative myocardial motion parameters and serum biomarker indexes after treatment (*P* < 0.05), indicating better clinical efficacy and lower recurrence rates. The 2C3L strategy involves circumferential bilateral pulmonary vein isolation (2C) and intraoperative ablation of MI, CTI, and LA roof lines (3L), while the 3C4L strategy encompasses left and right pulmonary vein adventitia isolation, superior vena cava isolation (3C), and MI, CTI, LA roof and floor lines (4L). The key addition in the 3C4L strategy is the posterior wall isolation line. The posterior wall of the left atrium constitutes an important component in AF maintenance, containing complex myocardial fiber arrangements and electrical conduction pathways prone to forming reentrant circuits. This finding aligns with previous work demonstrating the importance of posterior wall isolation (17).

The 3C4L strategy achieves more comprehensive isolation or destruction of potential ectopic triggering foci through expanded ablation scope, thereby promoting more homogeneous electrical conduction in the left atrium and reducing localized conduction delays and refractory loops (18). The additional ablation lines alter electrical conduction pathways and electrophysiological characteristics in the left atrium, promoting overall electrical conduction consistency and more orderly electrical activity propagation. This reduces continuous stretching and pressure elevation in the left atrium due to AF, enabling the atrium to maintain relatively normal systolic and diastolic functions while preventing further dilatation and remodeling. The strategy restores normal mechanical function of the atrium, including coordination of atrial systole and diastole, providing better protection and restoration of atrial mechanical function and helping maintain overall cardiac circulation, thereby improving myocardial exercise parameters.

The pathophysiology of persistent AF involves atrial stromal fibrosis with electrical and structural remodeling of the left atrium leading to electrical activity disturbances through multiple signaling pathways, inflammatory processes, and stress responses reflected in various serum indicators (19). Ablation reduces AF episodes and attenuates myocardial injury from disturbed electrical activity, decreasing myocardial cell damage, necrosis, and apoptosis, thereby lowering NT-ProBNP levels. It improves electromechanical disturbances within the atria, atrial blood perfusion, and tissue metabolism, which inhibits inflammatory cell activation and aggregation, normalizes inflammatory regulatory factor secretion by endothelial cells, and decreases MCP-1 release. Furthermore, ablation improves cardiac pumping function and renal perfusion, reducing cardiomyocyte energy metabolism disorders, attenuating oxidative stress, and decreasing UA production. The strategy also reduces inflammatory responses and atrial remodeling, thereby modulating the sialylation process of cell surface glycoproteins and decreasing Neu5Ac levels. Improved cardiac function attenuates intestinal stasis, repairs intestinal barrier function, and reduces intestinal bacterial translocation and production of prerequisite substances such as paracresol, thereby lowering IS levels (20–22).

This study demonstrated that the 3C4L ablation procedure had significantly longer total procedure time, MI ablation time, and X-ray exposure time and greater saline infusion volume compared to the 2C3L procedure (*P* < 0.05). However, the total complication rate did not differ significantly between groups (*P* > 0.05). The 3C4L strategy, like 2C3L, does not use AF termination as the ablation endpoint but rather maximizes bidirectional blockade of all ablation pathways (23). The significantly longer MI ablation time in the 3C4L group may be attributed to the complex anatomy of the MI region, where epicardial conduction pathways (e.g., ligament of Marshall) often exist, requiring longer duration to ensure complete block. High tissue impedance in this region may necessitate repeated ablation. In the 3C4L group, ethanol infusion into the vein of Marshall (EI-VOM) was performed when radiofrequency ablation achieved incomplete MI block. Alcohol infiltration into atrial myocardial tissue achieves transmural injury with persistent effects, reducing distant reconnection incidence and prolonging operation time (24). The significantly longer procedure time was mainly distributed in MI, SVCI, and LA floor line ablation. EI-VOM catheter positioning depends on X-ray guidance, requiring multiple adjustments in newly ablated areas, resulting in significantly increased total procedure time and X-ray exposure time (25). Compared to 2C3L, the 3C4L procedure adds more circumferential or linear ablations, requiring greater saline irrigation to cool the catheter tip and prevent tissue overheating (26).

The 3C4L strategy represents an integration of several ablation routes with proven feasibility and safety, supplemented by general anesthesia and guaranteed observation time, achieving a quantitative to qualitative transformation in radiofrequency ablation effectiveness for AF (27). While the 3C4L strategy extends the 2C3L approach and increases ablation scope, it does not fundamentally change the overall nature and type of involved areas. Although it theoretically increases operational complexity and prolongs procedure time, with mature surgical technique and relatively stable human anatomy, the complication rate difference between strategies is not significant (28).

This study has several limitations. First, the retrospective, non-randomized design introduces potential selection bias, as ablation strategy choice was determined by treating physicians based on temporal evolution of institutional practice and patient characteristics rather than randomization. This limits causal inference regarding treatment effects. Second, the relatively small sample size of 105 patients may not fully reflect the overall characteristics of the target population, potentially leading to insufficient statistical power to detect certain differences or Type II errors. Third, although 24-month follow-up provides valuable long-term data, extended follow-up would better characterize very late recurrences and durability of ablation effects. Fourth, the recovery process after radiofrequency ablation extends beyond the current observation period, and this study primarily focused on short-to-medium-term postoperative effects with limited assessment of very long-term outcomes in quantitative myocardial exercise parameters and serum biomarkers. Fifth, the study was conducted at a single center, which may limit generalizability to other institutions with different operator experience, patient populations, and procedural protocols. Finally, while Cox regression was performed to adjust for key confounders, unmeasured confounding factors may still influence the observed associations.

In conclusion, compared with the 2C3L radiofrequency ablation strategy, the 3C4L strategy demonstrates several advantages including superior clinical efficacy, better improvement of cardiac function, and significantly enhanced long-term arrhythmia-free survival, while not significantly increasing complication rates despite requiring longer procedure time. However, these findings should be interpreted with caution given the retrospective, non-randomized study design and potential selection bias inherent in treatment allocation. Prospective randomized controlled trials are needed to definitively establish the superiority of the 3C4L approach and to identify which patient subgroups would most benefit from this more extensive ablation strategy. Clinicians should consider individual patient characteristics, procedural risks, operator experience, and patient preferences when selecting the optimal treatment approach.

## Data Availability

The original contributions presented in the study are included in the article/Supplementary Material, further inquiries can be directed to the corresponding author.
